# Vancomycin-Associated Acute Kidney Injury: A Narrative Review from Pathophysiology to Clinical Application

**DOI:** 10.3390/ijms23042052

**Published:** 2022-02-12

**Authors:** Wei-Chih Kan, Yi-Chih Chen, Vin-Cent Wu, Chih-Chung Shiao

**Affiliations:** 1 Department of Nephrology, Department of Internal Medicine, Chi Mei Medical Center, Tainan 71004, Taiwan; rockiekan@ntu.edu.tw; 2 Department of Biological Science and Technology, Chung Hwa University of Medical Technology, Tainan 71703, Taiwan; 3 Department of Pharmacy, Camillian Saint Mary’s Hospital Luodong, Yilan 265, Taiwan; soarcloud@gmail.com; 4 Department of Internal Medicine, National Taiwan University Hospital, Taipei 100225, Taiwan; q91421028@ntu.edu.tw; 5 Division of Nephrology, Department of Internal Medicine, Camillian Saint Mary’s Hospital Luodong, Yilan 265, Taiwan; 6 Saint Mary’s Junior College of Medicine, Nursing and Management, Yilan 26647, Taiwan

**Keywords:** acute kidney injury, acute tubular necrosis, acute tubulointerstitial nephritis, allergic reaction, antibiotics, oxidative stress, vancomycin

## Abstract

Vancomycin is the most frequently used antibiotic, accounting for up to 35% of hospitalized patients with infection, because of its optimal bactericidal effectiveness and relatively low price. Vancomycin-associated AKI (VA-AKI) is a clinically relevant but not yet clearly understood entity in critically ill patients. The current review comprehensively summarizes the pathophysiological mechanisms of, biomarkers for, preventive strategies for, and some crucial issues with VA-AKI. The pathological manifestations of VA-AKI include acute tubular necrosis, acute tubulointerstitial nephritis (ATIN), and intratubular crystal obstruction. The proposed pathological mechanisms of VA-AKI include oxidative stress and allergic reactions induced by vancomycin and vancomycin-associated tubular casts. Concomitant administration with other nephrotoxic antibiotics, such as piperacillin–tazobactam, high vancomycin doses, and intermittent infusion strategies compared to the continuous infusion are associated with a higher risk of VA-AKI. Several biomarkers could be applied to predict and diagnose VA-AKI. To date, no promising therapy is available. Oral steroids could be considered for patients with ATIN, whereas hemodialysis might be applied to remove vancomycin from the patient. In the future, disclosing more promising biomarkers that could precisely identify populations susceptible to VA-AKI and detect VA-AKI occurrence early on, and developing pharmacological agents that could prevent or treat VA-AKI, are the keys to improve the prognoses of patients with severe infection who probably need vancomycin therapy.

## 1. Introduction

Acute kidney injury (AKI) is a common complicated disorder affecting about 2–7% of hospitalized patients and 13–78% of critically ill patients [1,2]. AKI can lead to increased morbidity and mortality [3]. The prognoses in AKI patients remain unsatisfactory despite advances in therapy for critically ill patients over the decades [4]. An essential strategy for resolving this disappointing situation is identifying the agents or factors that potentially cause or precipitate AKI in clinical practice to prevent subsequent AKI. The use of drugs is a modifiable risk factor for AKI and is responsible for around 18%–40% of AKI among critically ill patients [5], while antibiotics are crucial triggers of AKI among all drugs [6].

Vancomycin is a highly hydrophilic glycopeptide antibiotic with a molecular weight of 1450 Da. Vancomycin is the gold standard for treating methicillin-resistant Staphylococcus aureus (MRSA) and methicillin-resistant Staphylococcus epidermis infections. It also plays a bactericidal role against *Streptococcus* sp., *Enterococcus* sp., *Actinomyces* sp., *Clostridium* sp., and *Eubacterium* sp. [5]. Vancomycin binds to the acyl-D-Ala-D-Ala terminus and blocks the cross-linking of the peptidoglycan wall of the bacteria [7]. This action induces a series of processes and activates degradative enzymes that contribute to cell wall destruction and subsequent cell damage. Vancomycin’s optimal bactericidal effectiveness and relatively low price make it the most frequently prescribed antibiotic, and it accounts for up to 35% of hospitalized patients with infections [8,9]. The adverse effects of intravenous vancomycin administration include hypotension, dizziness, muscle pain or spasms, wheezing or shortness of breath, allergic reaction, phlebitis, and nephrotoxicity.

Vancomycin-associated AKI (VA-AKI) was first identified in 1958 [10]. Several randomized clinical trials subsequently found that vancomycin is associated with a higher AKI risk than most other antibiotics [11]. A systemic review and meta-analysis by Ray et al. that included 4033 patients found that vancomycin administration has leads to a 2.5-fold increased AKI risk [11]. VA-AKI often develops after 4–17 days of vancomycin therapy and improves after prompt discontinuation of vancomycin [6,12]. However, some patients, especially those with critical illness, do not experience full renal function recovery [6,12]. VA-AKI is associated with a prolonged hospital stay, hospital readmission rate, and patient mortality [13].

Clinically, laboratory findings and clinical judgment are essential for diagnosing VA-AKI [14]. However, a kidney biopsy may be necessary if the clinical diagnosis is equivocal [15] because only 59% of AKI patients treated with vancomycin are categorized as VA-AKI patients [11]. The reported incidence of VA-AKI ranges from 5% to 43% according to various clinical settings and AKI definitions. More recently, many investigators recommend diagnosing VA-AKI using existing AKI criteria, such as RIFLE, AKIN, and KDIGO criteria [11,13,16]. Nevertheless, the coexistence of infections and other risk factors for AKI in most reported cases makes it hard to evaluate the true incidence rate of VA-AKI.

VA-AKI is a clinically relevant entity. VA-AKI’s pathophysiological mechanisms and diagnostic and therapeutic strategies are not yet well clarified [12], although an increasing body of evidence regarding these issues has been continuously published until now. The current review comprehensively summarizes the pathophysiological mechanism, biomarkers, therapeutic strategies, and some crucial issues of VA-AKI.

## 2. Pharmacokinetics and Pharmacodynamics of Vancomycin

Vancomycin’s pharmacokinetics are recognized as a two-compartment (central and peripheral compartments) model with a biphasic (alfa and beta phases) fashion of drug concentration elimination [17]. The antibacterial effect of vancomycin is time- and concentration-dependent. Vancomycin has a volume of distribution of 0.4–1.0 L/kg in healthy subjects, which might increase up to two- to three-fold in critically ill patients [17]. In patients with normal creatinine clearance, vancomycin’s distribution phase lasts 30 min to 1 h, and the half-life elimination takes 6–12 h [18]. Vancomycin’s protein binding rate ranges from 10% to 64%, depending on the total serum proteins levels [19]. Vancomycin penetrates most tissues and has a high concentration in the kidneys, which is higher than in plasma [20].

Glomerular filtration excretes vancomycin with 80–90% non-metabolized form into the lumen of the proximal tubule, followed by minimal reabsorption and metabolism by proximal tubular cells [21,22]. On the other hand, vancomycin is also actively transported from the peritubular circulation into proximal tubule cells by the organic cation transporter (OCT)-2, which is located at the basolateral membrane of the tubular cell, and then secreted into the tubular lumen from the apical membrane of the proximal tubule by an efflux transporter, P-glycoprotein [23,24]. Subsequently, vancomycin is transported from the tubular lumen into the tubular cells across the apical membrane by apical endocytosis through dehydropeptidase (DHP)-1 and megalin. In addition, the vancomycin-mediated inhibition of the expression and function of P-glycoprotein also promotes vancomycin accumulation in the tubular cells. The high vancomycin concentration at the brush border is supporting evidence for the ongoing secretion to the lumen, or the reabsorption from the lumen of vancomycin at the apical membrane. In brief, vancomycin enters the tubular epithelial cells via receptor-mediated endocytosis from the urine, and via transporter-mediated secretion from the peritubular circulation [25]. Both pathways cause the drug to accumulate in the tubular epithelial cells’ cytoplasm and expose the tubular cells and the surrounding interstitium to potentially nephrotoxic substances [26]. Hongjing et al. [20] demonstrated that a high dose of vancomycin administration resulted in the induction of many drug transporters of proximal tubular cells in an animal model. These drug transporters include those located in the basolateral membrane (e.g., organic anion transporter (OAT)-1, OAT-3, OCT-2) and those located at the apical membrane (breast cancer resistance protein (BCRP), multidrug and toxin extrusion protein (MATE)-1, MATE-2k, multidrug resistance protein (MRP)-2, and MRP-4) [20]. The association between the alternation of kidney transporters and the kinetic process of VA-AKI has not been fully clarified, but it has crucial clinical implications necessitating further evaluation [20].

## 3. Pathological Manifestations of VA-AKI

VA-AKI has several pathological manifestations that include acute tubular necrosis (ATN), acute tubulointerstitial nephritis (ATIN), and intratubular crystal obstruction [6]. The exact prevalence of these pathological manifestations in VA-AKI patients is unknown because of the low percentage of kidney biopsies among patients who receive vancomycin therapy and have AKI.

Recently, Tantranont et al. [14] systematically reviewed kidney biopsy specimens of AKI patients who received vancomycin treatment between 2010 and 2019 in the Houston Methodist Hospital System. Among the total thirty-seven enrolled patients, twenty-five patients (67.6%) had both ATN and ATIN, five patients (13.5%) had ATN alone, three patients (8.1%) had acute or chronic tubulointerstitial nephritis (TIN) alone, and four patients (10.8%) had interstitial fibrosis and tubular atrophy. In addition, Bellos et al. [27] systematically summarized and analyzed 21 patients from 18 case reports or case series reporting on individual patients with biopsy-proven VA-AKI published from 1989 to September 2020. The authors reported that three patients (14.3%) had both ATN and ATIN, ten patients (47.6%) had ATN alone, and nine patients (42.9%) had ATIN alone. In summary, both studies reported that the first two predominant histological patterns of VA-AKI were ATN (accounting for 61.9% to 81.1%) and ATIN (57.1% to 75.7%) [14,27].

Furthermore, Bellos et al. [27] applied time-to-event analysis to demonstrate that ATIN carried a five-fold higher risk of permanent renal dysfunction than ATN, and interstitial fibrosis was associated with a considerably worse renal prognosis.

## 4. Pathophysiological Mechanisms of VA-AKI

**Figure 1:**
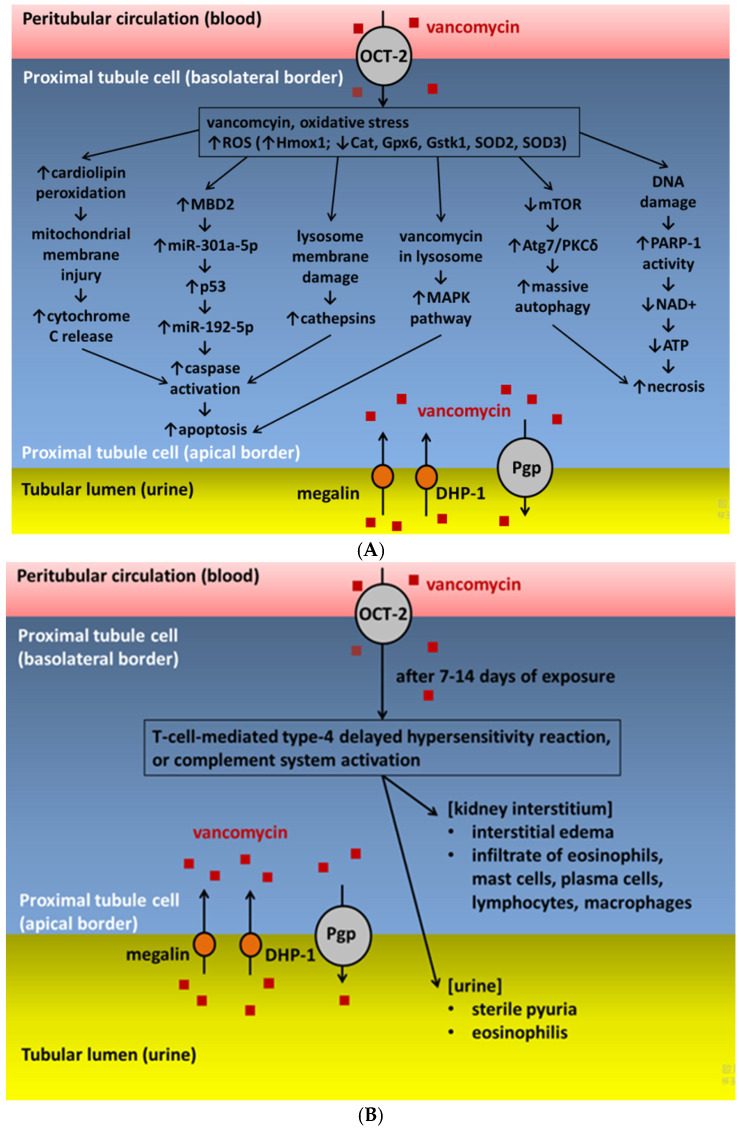

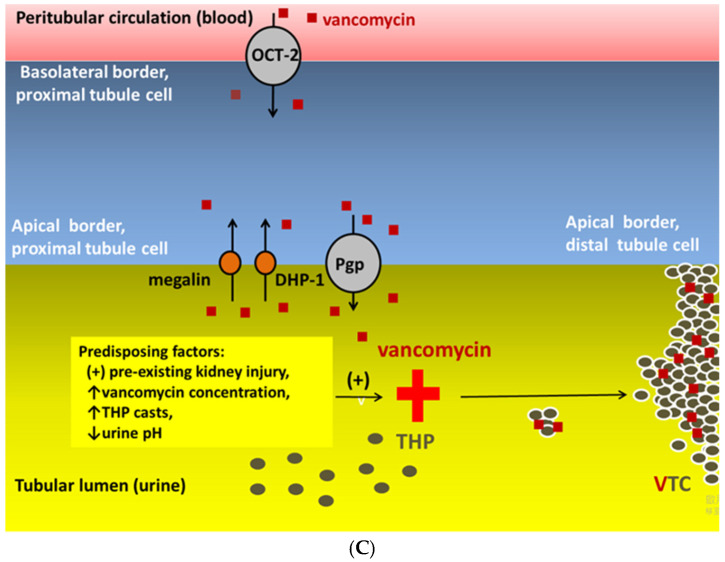
Pathophysiological mechanisms of VA-AKI associating with (**A**) oxidative stress, (**B**) allergic reaction, and (**C**) vancomycin-associated tubular casts. Abbreviations: Atg, autophagy-related gene; ATP, adenosine triphosphate; Cat, catalase; DHP-1, dehydropeptidase-1; DNA, deoxyribonucleic acid; Gpx6, glutathione peroxidase 6; Gstk1, glutathione S-transferase kappa 1; Hmox1, heme oxygenase 1; MAPK, mitogen-activated protein kinase; MBD2, methyl-CpG-binding domain protein 2; miR, microRNA; mTOR, mammalian target of rapamycin; NAD, nicotinamide adenine dinucleotide; OCT-2, organic cation transporter 2; PARP-1, poly (adenosine diphosphate ribose) polymerase 1; Pgp, P-glycoprotein; PKCδ, protein kinase C delta; ROS, reactive oxygen species; SOD, superoxide dismutase; THP, Tamm–Horsfall glycoprotein.

The exact pathophysiological mechanisms of VA-AKI are not yet fully understood. However, the existing consensus states that vancomycin-induced proximal tubular epithelium is directly caused by intracellular accumulation of the drug when it is endocytosed into the tubular cells [21,28,29]. The main mechanisms of VA-AKI include high intracellular drug concentration in the renal tubules of individuals with risk factors for nephrotoxicity, subsequently inducing oxidative stress, complement activation, inflammatory injury, mitochondrial dysfunction, and cellular apoptosis in proximal renal tubules [14]. Figure 1 summarizes the pathophysiological mechanisms of VA-AKI.

### 4.1. Oxidative Stress

Oxygen consumption in cells generates reactive oxygen species (ROS) [30]. Oxidative stress is an imbalance between ROS and antioxidants within cells. Oxidative stress leads to mitochondrial dysfunction and cellular apoptosis and is the primary mechanism of VA-AKI [31]. When vancomycin enters proximal tubular cells, it stimulates oxidative phosphorylation, increasing oxygen consumption and adenosine triphosphate (ATP) concentration, generating iron complex and subsequent ROS. A recent human study demonstrated that lower serum serotonin (5-HT), higher serum 5-hydroxy indole acetic acid (5-HIAA), and a higher ratio of 5-HIAA to 5-HT was associated with the occurrence of VA-AKI. The finding suggests that the high 5-HIAA/5-HT ratio could be a potential surrogate biomarker for VA-AKI, and indicates that acute oxidative stress and inflammation are involved in in the mechanism of VA-AKI [32].

Figure 1A summarizes the mechanisms associated with oxidative stress for VA-AKI. The ROS upregulates the transcriptional expression of the Hmox1 gene and methyl-CpG-binding domain protein 2 (MBD2). The Hmox1 gene is an indicator of cellular oxidative stress that downregulates the expression of catalase (Cat), glutathione peroxidase 6 (Gpx6), glutathione S-transferase kappa 1 (Gstk1), superoxide dismutase (SOD)-2, and SOD-3, which encode some primary cellular antioxidants [28].

ROS induce lipid peroxidation that affects cardiolipin in cell membranes, resulting in mitochondrial membrane depolarization [26]. The mitochondrial membrane damage subsequently induces cytochrome C release, caspases activation, and cell apoptosis [33]. Vancomycin causes a dose-dependent increase in ATP concentrations in cultured renal cells [34], which indicates that vancomycin can stimulate mitochondrial oxidative phosphorylation [23]. Additionally, the increased oxygen consumption and ROS production cause injury to the mitochondrial deoxyribonucleic acid (DNA) [6]. Mitochondrial ROS is an endogenous inducer of DNA single-strand breaks that promote DNA damage and cause the activation of poly-adenosine diphosphate ribose polymerase 1 (PARP-1), an enzyme involved in DNA repair [35]. The PARP-1 utilizes nicotinamide adenine dinucleotide (NAD+) as a substrate during the DNA repair, and cells consume ATP to refill NAD+ stores. As a result, PARP-1 activity overactivation following high amounts of DNA damage subsequently causes a depletion of NAD+/ATP and cell necrosis. Several findings support the roles of PARP-1 in VA-AKI. This evidence includes the overactive PARP-1 activity in rats administered with vancomycin, the attenuated kidney injury after treatment with a PARP inhibitor, and the reversed kidney histopathological damage after the administration of antioxidants [23]. A high ROS concentration also activates MBD2, a protein reader of methylation. MBD2 activates the microRNA (miR)-301a-5p that subsequently upregulates p53 and activates miR-192–5p, which induces caspase activation, resulting in apoptosis [5,36].

Vancomycin accumulates in lysosomes because endosomes entrap vancomycin and then fuse with lysosomes [24]. The accumulation of vancomycin in lysosomes activates the mitogen-activated protein kinase (MAPK) pathway, resulting in programmed cell death or the apoptosis of the proximal tubular cells [6,22,28]. A study demonstrated a reduction of vancomycin’s nephrotoxic effect when patients were prescribed a β-glucuronidase inhibitor [29]. This finding suggested that lysosomal enzymes may activate vancomycin’s nephrotoxic effect. In addition, vancomycin suppresses ERK1/2/mammalian target of rapamycin (mTOR) activation to enhance the interaction of autophagy-related gene (Atg) 7 and protein kinase C delta (PKCδ), and then subsequently mediates increased autophagy in kidney cells and tissues [37]. Although autophagy performs a self-degradation process of the cellular components that play a protective role against AKI [38], massive autophagy induced by vancomycin may cause cell death, resulting in VA-AKI. This mechanism is supported by the finding that VA-AKI was attenuated in the proximal tubule-specific Atg7-knockout mouse model in which autophagy was inhibited [37].

The production of ROS may also induce the permeabilization of lysosomes that are near the mitochondria. Lysosomal membrane damage releases proteases, such as cathepsins, into the cytosol that activate apoptotic effectors, such as the mitochondria and caspases. The complete disruption of lysosomes may induce cytosolic acidification, which in turn provokes uncontrolled cell death by necrosis [39]. In addition, genetic polymorphisms in the OCTs predispose the kidney to injury after drugs exposure. All the above mechanisms contribute to ATN in VA-AKI (Figure 1A). ATN is processed in a dose-dependent manner and is the primary clinical manifestation of VA-AKI. It mainly occurs in patients with risk factors for renal injury [40]. During the cell damage process, the renal proximal tubular epithelium undergoes a loss of cytoskeletal integrity, necrosis, and apoptosis [41]. Necrotic cells release molecules that upregulate the innate immune system, inducing inflammation and accelerating tubular injury [41]. In addition, the urinary inflammatory proteins are upregulated in patients with VA-AKI [12].

### 4.2. Allergic Reaction

Allergic reaction is another possible mechanism involved in VA-AKI [42]. The supporting evidence includes the ATIN with significant eosinophil infiltration found in some kidney biopsy specimens of patients with VA-AKI [15], and a reported case with recurrent ATIN after a secondary challenge of vancomycin [43]. ATIN is a dose-independent acute idiosyncratic inflammatory condition involving renal tubules and interstitium [40,44]. Drug-induced ATIN accounts for 3–15% of all drug-induced AKI [40] and 60–70% of all ATIN cases [44]. However, drug-induced ATIN is difficult to diagnose since it occurs after a widely varied period, usually 7–14 days, following drug exposure, and the typical triad of symptoms, including fever, rashes, and eosinophilia, is only revealed in 10% of the patients. The manifestations of ATIN include sterile pyuria, eosinophiluria, and characteristic presentations on kidney biopsy specimens, including interstitial edema with infiltrations of eosinophils, mast cells, plasma cells, lymphocytes, and macrophages in the renal interstitium [45]. In most cases, ATIN is self-limited, and kidney function might recover after a period of weeks to months.

Although the mechanism of vancomycin-induced AIN is not yet completely understood, it is proposed to be associated with the T-cell-mediated type-4 delayed hypersensitivity reaction [40,45] or probable complement system activation [12]. (Figure 1B) In an animal study, the altered expression of several transcripts from the complement system in kidney tissue was demonstrated in mice receiving high-dose vancomycin. This complement included complement component 3 (C3), complement component 4b (C4b), and C-X-C motif chemokine ligand 1 (Cxcl1), which is a biomarker of ischemic kidney injury produced in a complement-dependent fashion [46]. Nevertheless, the role of the complement pathway in VA-AKI needs further elucidation.

### 4.3. Vancomycin-Associated Tubular Cast

In the recent study by Tantranont et al. [14] that evaluated the pathological manifestations of VA-AKI, 28 of the 37 patients who received vancomycin and developed AKI (75.7%) were diagnosed with VA-AKI by definition, including the characteristic light microscopic findings and with a renal function recovery after vancomycin discontinuation. VTC was disclosed in 25 of the 28 (89.3%) VA-AKI patients, but only one of the nine patients (11.1%) was without VA-AKI. The existence of VTCs is associated with a background of more diffuse renal injury [14].

The VTCs are casts composed of vancomycin aggregates and Tamm–Horsfall glycoproteins (THP) that are predisposed with a high urine vancomycin concentration and a low urine pH (<5.5) [28]. The VTCs mainly localize in the distal tubules [14,27], block the urine flow in distal tubules, trigger inflammation of the surrounding interstitium, and consequently cause AKI [26].

A hypothesis underscores the importance of an initial insult that creates de novo or accentuates pre-existing kidney injury. This insult subsequently causes THP casts, the increased local concentration and precipitation of vancomycin, and the localized necrosis of tubular epithelial cells [14]. An increasing body of evidence indicates that VTCs have a nephrotoxic effect superimposing on and independent from the ATN or AIN in the pathogenesis of VA-AKI. Thus, VTC is considered to be a new mechanism of VA-AKI and a characteristic morphologic profile facilitating the biopsy diagnosis of VA-AKI [14,27]. (Figure 1C).

## 5. Some Crucial Issues Regarding VA-AKI

### 5.1. Concomitant Administration with Piperacillin–Tazobactam

In animal studies, it is known that additional nephrotoxic agents additively or synergistically increase vancomycin-mediated nephrotoxicity [28]. Clinically, the combination therapy of vancomycin and other antibiotics is often prescribed in critically ill patients infected with multiple drug-resistant pathogens. The combination of vancomycin and piperacillin–tazobactam is among the most often prescribed antibiotic therapies in the hospital setting [47]. Previous clinical studies found that piperacillin–tazobactam carries a significantly higher risk of AKI than other comparator antibiotics when concurrently prescribed with vancomycin [16]. Although conflicting results exist [48,49], similar findings were reported in some review work, a meta-analysis [50], and a network meta-analysis [51].

The mechanism of increasing AKI risk with the combination of vancomycin and piperacillin–tazobactam is still unclear [50], but several potential supporting pieces of evidence have been proposed. First, piperacillin–tazobactam itself is possibly a nephrotoxic agent. Several investigations disclosed that piperacillin–tazobactam monotherapy is associated with an increased AKI risk even higher than vancomycin monotherapy [52], and piperacillin–tazobactam has shown the lowest renal recovery rate among beta-lactams antibiotics [53]. Second, piperacillin–tazobactam is associated with interstitial nephritis [54]. Third, piperacillin–tazobactam decreases vancomycin clearance and elevates plasma vancomycin concentration [55]. Fourth, the sodium content may increase the AKI risk of the combination of piperacillin–tazobactam and vancomycin. Fifth, since OAT-1 and OAT-3 mediate creatinine transit from peritubular circulation to tubular cells, the combined use of vancomycin and piperacillin–tazobactam synergistically competes with the creatinine on OATs and MATEs, causing the accumulation of serum creatinine. Moreover, vancomycin suppresses the miRNA and protein expressions of OAT-1 and OAT-3, thus enhancing the degree of creatinine accumulation [56]. This hypothesis might be supported by the more prompt recovery of AKI associated with piperacillin–tazobactam than other antibiotics [53].

### 5.2. Administration Dosage and VA-AKI

It is well known that VA-AKI is directly caused by a higher intracellular accumulation of the drug when vancomycin enters into the tubular cells [21,28,29]. In addition, a high dose of vancomycin administration induces the activity of many drug transporters at proximal tubular cells, increasing the vancomycin concentration in tubular cells [20]. Thus, it is evident that dosage matters in causing nephrotoxicity. Animal studies disclosed that a higher vancomycin dose and longer treatment duration in rats were associated with increased histopathological damage and elevations in the urinary biomarkers of AKI [57]. Studies in humans also found an association between a higher vancomycin dosage and a higher AKI risk [58]. A meta-analysis of eight observational studies including 2491 patients demonstrated that an area under the curve (AUC) of less than 650 mg × h/L was associated with a decreased risk of VA-AKI. The odds ratios (ORs) (95% confidence interval (CI)) were 0.36 (0.23–0.56) and 0.45 (0.27–0.75) when measuring the vancomycin exposure in the first 24 h (AUC0–24) or second 24 h (AUC24–48), respectively [58].

### 5.3. Administration Patterns and VA-AKI

Flannery et al. [59] conducted a systematic review and meta-analysis, enrolling 11 studies involving 2123 patients, to investigate the impact of vancomycin infusion strategy on AKI in critically ill adults. The study found that continuous infusion was associated with a 53% lower AKI risk than the intermittent infusion strategy in critically ill adults [59]. This is potentially because the continuous infusion method causes less risk of high peak vancomycin concentrations than intermittent infusion, and the maximum level and AUC of vancomycin during the therapeutic interval are most linked to AKI [60].

Besides the safety issue, the continuous infusion of vancomycin might be more beneficial than intermittent infusion administration in several ways. These reasons include a lower requirement and cost for drug concentration monitoring, a more straightforward dosing adjustment method, and a better AUC/minimum inhibitory concentration (MIC) and pharmacokinetic target attainment over the dosing interval with less variability [59].

Nevertheless, some crucial issues potentially prohibit the broad adoption of the continuous infusion of vancomycin in critical care settings. First, the continuous infusion of vancomycin was associated with more endothelial cell toxicity than intermittent infusion administration [61]. Second, continuous drug administration has a higher requirement for the availability of intravenous access than intermittent infusion. Thus, the continuous infusion of vancomycin is suggested to be given via a central line or, if necessary, by peripheral administration at a lower concentration. The meta-analysis had some limitations regarding the enrolled studies, such as the biases of observational research and the limited patient number of the two enrolled randomized control trials. Nevertheless, continuous infusion should be considered to be a potential benefit for lowering the VA-AKI risk after assessing patients’ clinical situations.

## 6. Biomarkers for Detecting VA-AKI

Experimental studies have demonstrated that vancomycin has a strong exposure–response correlation with increased kidney histopathological damage [57] and elevations in the urinary AKI biomarkers of AKI [57]. Moreover, the histopathological damage is correlated with the changes in urinary biomarkers [57].

Table 1 lists the proposed biomarkers for predicting VA-AKI. An experimental study enrolling 125 rats demonstrated that urinary kidney injury molecule-1 (KIM-1) and urinary clusterin were the most sensitive biomarkers for predicting the earliest injury after 24 hrs of vancomycin treatment, including low-level histopathological damages and moderate-level histopathological damages. Urinary KIM-1 and urinary clusterin were also the best biomarkers for predicting moderate-level histopathological damage at day 3 (AUC 0.82 (95% CI 0.70–0.95), *p* = 0.037 and 0.801 (0.67–0.93), *p* = 0.060, respectively) and day 6 (AUC 0.91 (0.79–1.00) and 0.86 (0.74–1.00), respectively), while urinary osteopontin was also a good biomarker for predicting VA-AKI [62].

In a human study, Pang et al. [63] conducted a prospective study enrolling 87 adult patients who received vancomycin therapy. The study disclosed that urinary KIM-1 and neutrophil gelatinase-associated lipocalin (NGAL) were promising biomarkers in discriminating between patients with and without VA-AKI earlier than serum creatinine [63]. Another two urinary biomarkers, tissue inhibitor of metalloproteinases 2 (TIMP-2) and insulin-like growth factor-binding protein 7 (IGFBP-7), were also associated with VA-AKI and adverse events within nine months in a prospective multicenter study enrolling 333 critically ill adult patients [65]. The [TIMP-2] × [IGFBP-7] value obtained on day 1 of vancomycin administration was independently associated with VA-AKI (*p* < 0.001) [65]. A prospective cohort study of 94 patients receiving vancomycin found that the urinary NGAL level between 96 and 144 hrs (OR 1.123, 95%CI 1.096–1.290, *p* = 0.03) was a predictor of AKI development, whereas a higher urinary [TIMP-2]x[IGFBP-7]/Cr between 144 and 192 hrs (OR 1.26, 95%CI 1.092–1.543, *p* = 0.03) was a predictor of non-recovery of VA-AKI [64]. In addition, a recent study by Awdishu et al. [12] found that urinary concentrations of some inflammatory proteins (namely, complement C3, C4, galectin-3-binding protein, fibrinogen, alpha-2 macroglobulin, immunoglobulin heavy constant mu, and serotransferrin) increased after nephrotoxic injury in patients with VA-AKI. These findings also suggest that the pathophysiology of VA-AKI is tubular toxicity with the upregulation of inflammation during the 24–72 h following injury [12].

As mentioned earlier, a high 5-HIAA/5-HT ratio in serum (AUC 0.88 (0.88–0.96)) was proposed as a potential surrogate biomarker for VA-AKI in humans [32]. Furthermore, Kim et al. retrospectively compared serum biomarkers among patients with and without VA-AKI. They found that serum cystatin C (AUC 0.92), trefoil factor-3 (AUC 0.93), tumor necrosis factor receptor 1 TNF-R1 (AUC 0.87), and osteopontin (AUC 0.79) exhibited excellent to outstanding diagnostic abilities for VA-AKI [66].

## 7. Potential Risk Factors Associated with VA-AKI

Table 2 summarizes the potential patient-related and treatment-related risk factors associated with VA-AKI [6,21,25,26,40,50,54,67,68,69,70]. The modifiable patient-related risk factors include intravascular volume depletion and concurrent acute illness, such as AKI, systemic infection/inflammatory, and electrolyte and acid–base disturbances. In addition, several non-modifiable patient-related risk factors include older age, female gender, race, an allergic response to drugs, altered pharmacogenetics, and pre-existing systemic comorbidities (Table 2).

## 8. Treatment of VA-AKI

To date, no promising therapy is available to treat VA-AKI. The only exception is that several case series suggest that the treatment of biopsy-proven ATIN with oral steroids for four weeks (prednisone, 1 mg/kg/day) may accelerate the rate of recovery [71]. Since some kidney injuries caused by vancomycin are generally idiosyncratic, management involves the removal of the suspected causative agent and supportive therapy. The prompt removal of vancomycin is the critical management solution for patients with suspected VA-AKI. Hemodialysis, particularly hemodialysis using a high-flux filter, is a helpful method to remove vancomycin from the patients [72]. High-flux hemodialysis filters, including polysulfone and polymethylmethacrylate, can remove vancomycin from patients more effectively (35–46%) [73]. However, frequent hemodialysis might be necessary for better therapeutic results because the plasma vancomycin concentration rebounds 3–6 h after a hemodialysis session [73].

## 9. Preventive Strategies for VA-AKI

Since the conventional treatment for VA-AKI is still limited, prevention is currently the most crucial strategy. The first step of the preventive strategy for VA-AKI is risk stratification according to the known risk factors listed in Table 2. Physicians might consider prescribing alternative antibiotics instead of vancomycin for the patients categorized as high-risk patients for VA-AKI. The alternative antibiotics for treating MRSA infection include teicoplanin, linezolid, daptomycin, tigecycline, and ceftaroline. Moreover, antibiotic susceptibility testing-based de-escalation with cefazolin or oxacillin is an appealing strategy to avoid the risk of VA-AKI.

For those who have to receive vancomycin therapy, the preventive management includes: (1) adequate hydration before and during the vancomycin treatment course for the patients with intravascular volume depletion; (2) treatment and correction of the concurrent acute illness of the patients; (3) avoiding the concomitant use of other potentially nephrotoxic agents if other choices of drugs exist; (4) using the continuous infusion method rather than the intermittent infusion method; (5) avoiding treatment durations in excess of seven days if possible; (6) avoiding excessive vancomycin exposure by therapeutic drug monitoring and dosage adjustment (the updated guidelines recommend an AUC/MIC ratio of 400–600 (assuming a vancomycin MIC of 1 mg/L) as the target for patients with severe MRSA infections for considering both clinical efficacy and patient safety [70]); (7) considering promptly de-escalating vancomycin dose or shifting to alternative drugs for those who are excluded from having severe MRSA infection, who are recognized as having had excessive vancomycin exposure, or who are found to have AKI [70,74].

## 10. Conclusions

In conclusion, VA-AKI is a clinically relevant but not yet clearly understood entity among critically ill patients. An increasing body of evidence regarding its pathophysiological mechanism, risk, and preventive strategies has been continuously published up to now. The pathological mechanisms of VA-AKI include oxidative stress induced by vancomycin, allergic reactions to vancomycin, and VTCs. All these mechanisms cause ATN, ATIN, and VTCs. 

In the future, disclosing more promising biomarkers that could precisely identify susceptible populations for VA-AKI and detect VA-AKI occurrence early, and developing pharmacological agents that could prevent or treat VA-AKI, are the keys to improve the prognoses of patients with severe MRSA infection who probably need vancomycin therapy.

## Figures and Tables

**Table 1:** Proposed biomarkers of VA-AKI.

Biomarkers (Specimen Source)	Subjects	AUC (95%CI)	Cut-off Value/ Sensitivity (%)/ Specificity (%)	References
Animal Studies				
KIM-1 (urine)	125 rats	0.82 (0.70–0.95) *p* = 0.037 *	6.11 ng/mL 83.8%/89.8%	Pais, 2019 [62]
Clusterin (urine)	125 rats	0.80 (0.67–0.93) *p* = 0.060 *	----	Pais, 2019 [62]
Osteopontin (urine)	125 rats	0.70 (0.53–0.86) *	----	Pais, 2019 [62]
Human studies				
KIM-1 (urine)	87 patients	0.85 (0.75–0.95) *p* < 0.001 **	1.72 ng/mL 81.8%/85.5%	Pang, 2017 [63]
NGAL (urine)	87 patients	0.82 (0.73–0.92) *p* = 0.001 **	9.07 ng/mL 100.0%/63.2%	Pang, 2017 [63]
KIM-1 +NGAL (urine)	87 patients	0.85 (0.75–0.95) *p* < 0.001 **	1.72 ng/mL (KIM-1) and 9.07 ng/mL (NGAL); 90.9%/75.0%	Pang, 2017 [63]
NGAL (urine) at 96–144 hr	94 patients	0.82 (0.61–0.96), *p* = 0.020 (for predicting VA-AKI by day 5)	618.8 ng/mL 73.0%/68.0%	Sampaio, 2021 [64]
[TIMP-2] × [IGFBP-7] (urine)	333 patients	----	----	Kane-Gill, 2019 [65]
[TIMP-2] × [IGFBP-7]/Cr at 144–192 h (urine)	94 patients	0.71 (0.62–0.98), *p* = 0.009 (for predicting non-recovery of VA-AKI at discharge)	2.15 (ng/mL)2/1000 88.0%/64.0%	Sampaio, 2021 [64]
5-HIAA/5-HT ratio (serum)	97 patients	0.88 (0.88–0.96)	----	Lee, 2021 [32]
Cystatin C (serum)	73 patients	0.92	----	Kim, 2021 [66]
Osteopontin (serum)	73 patients	0.79	----	Kim, 2021 [66]
TFF3 (serum)	73 patients	0.93	----	Kim, 2021 [66]
TNF-R1 (serum)	73 patients	0.87	----	Kim, 2021 [66]

Note: * biomarkers obtained on day 3 for predicting moderate-level histopathological damage at day 3, ** biomarkers obtained on day 2—data not provided from the cited studies. Abbreviations: 5-HIAA, 5-hydroxy indole acetic acid; 5-HT, serotonin; AUC, area under the curve; CI, confidence interval; Cr, creatinine; IGFBP-7, insulin-like growth factor-binding protein 7; KIM-1, kidney injury molecule 1; NGAL, neutrophil gelatinase-associated lipocalin; OR, odds ratio; TFF3, trefoil factor-3; TIMP-2, tissue inhibitor of metalloproteinases 2; TNF-R1, tumor necrosis factor receptor 1.

**Table 2:** Potential risk factors associated with VA-AKI.

	Potential Risk Factors
Modifiable	Effective intravascular volume depletion Concurrent acute illness: acute kidney injury or acute kidney disease, systemic infection/inflammation, hypotension, immunosuppression state, increased disease severity, electrolyte, and acid–base disturbances
Non-modifiable	Older age Female gender Race Allergic response to drugs Altered pharmacogenetics (kidney drug transporters, cytochrome P450 enzyme gene polymorphisms) Pre-existing systemic comorbidities: chronic kidney disease, nephrotic syndrome, advanced liver cirrhosis, obstructive jaundice, cardiovascular comorbidities (including heart failure), diabetes mellitus, obesity, immunosuppression state

## Data Availability

Not applicable.

## References

[1] Mas-Font S., Ros-Martinez J., Perez-Calvo C., Villa-Diaz P., Aldunate-Calvo S., Moreno-Clari E. (2017). Prevention of acute kidney injury in Intensive Care Units. Med. Intensiva.

[2] Hoste E.A., Bagshaw S.M., Bellomo R., Cely C.M., Colman R., Cruz D.N., Edipidis K., Forni L.G., Gomersall C.D., Govil D. (2015). Epidemiology of acute kidney injury in critically ill patients: The multinational AKI-EPI study. Intensive Care Med..

[3] Wu V.C., Huang T.M., Lai C.F., Shiao C.C., Lin Y.F., Chu T.S., Wu P.C., Chao C.T., Wang J.Y., Kao T.W. (2011). Acute-on-chronic kidney injury at hospital discharge is associated with long-term dialysis and mortality. Kidney Int..

[4] Druml W. (2014). Systemic consequences of acute kidney injury. Curr. Opin. Crit. Care.

[5] Petejova N., Martinek A., Zadrazil J., Kanova M., Klementa V., Sigutova R., Kacirova I., Hrabovsky V., Svagera Z., Stejskal D. (2020). Acute kidney injury in septic patients treated by selected nephrotoxic antibiotic agents-pathophysiology and biomarkers-a review. Int. J. Mol. Sci.

[6] Morales-Alvarez M.C. (2020). Nephrotoxicity of antimicrobials and antibiotics. Adv. Chronic. Kidney Dis..

[7] Kim S.J., Matsuoka S., Patti G.J., Schaefer J. (2008). Vancomycin derivative with damaged D-Ala-D-Ala binding cleft binds to cross-linked peptidoglycan in the cell wall of Staphylococcus aureus. Biochemistry.

[8] Mitevska E., Wong B., Surewaard B.G.J., Jenne C.N. (2021). The prevalence, risk, and management of methicillin-resistant staphylococcus aureus infection in diverse populations across Canada: A systematic review. Pathogens.

[9] Diallo O.O., Baron S.A., Abat C., Colson P., Chaudet H., Rolain J.M. (2020). Antibiotic resistance surveillance systems: A review. J. Glob. Antimicrob. Resist..

[10] Geraci J.E., Heilman F.R., Nichols D.R., Wellman W.E. (1958). Antibiotic therapy of bacterial endocarditis. VII. Vancomycin for acute micrococcal endocarditis; preliminary report. Proc. Staff Meet. Mayo Clin..

[11] Sinha Ray A., Haikal A., Hammoud K.A., Yu A.S. (2016). Vancomycin and the risk of AKI: A systematic review and meta-analysis. Clin. J. Am. Soc. Nephrol..

[12] Awdishu L., Le A., Amato J., Jani V., Bal S., Mills R.H., Carrillo-Terrazas M., Gonzalez D.J., Tolwani A., Acharya A. (2021). Urinary exosomes identify inflammatory pathways in vancomycin associated acute kidney injury. Int. J. Mol. Sci..

[13] Jorgensen S.C.J., Murray K.P., Lagnf A.M., Melvin S., Bhatia S., Shamim M.D., Smith J.R., Brade K.D., Simon S.P., Nagel J. (2020). A multicenter evaluation of vancomycin-associated acute kidney injury in hospitalized patients with acute bacterial skin and skin structure infections. Infect. Dis. Ther..

[14] Tantranont N., Luque Y., Hsiao M., Haute C., Gaber L., Barrios R., Adrogue H.E., Niasse A., Truong L.D. (2021). Vancomycin-associated tubular casts and vancomycin nephrotoxicity. Kidney Int. Rep..

[15] Tantranont N., Obi C., Luque Y., Truong L.D. (2019). Vancomycin nephrotoxicity: Vancomycin tubular casts with characteristic electron microscopic findings. Clin. Nephrol. Case Stud..

[16] Kunming P., Can C., Zhangzhang C., Wei W., Qing X., Xiaoqiang D., Xiaoyu L., Qianzhou L. (2021). Vancomycin associated acute kidney injury: A longitudinal study in China. Front. Pharmacol..

[17] Zamoner W., Prado I.R.S., Balbi A.L., Ponce D. (2019). Vancomycin dosing, monitoring and toxicity: Critical review of the clinical practice. Clin. Exp. Pharmacol. Physiol..

[18] DiMondi V.P., Rafferty K. (2013). Review of continuous-infusion vancomycin. Ann. Pharmacother..

[19] Butterfield J.M., Patel N., Pai M.P., Rosano T.G., Drusano G.L., Lodise T.P. (2011). Refining vancomycin protein binding estimates: Identification of clinical factors that influence protein binding. Antimicrob. Agents Chemother..

[20] Li H., Yang Q., Gui M., Ding L., Yang L., Sun H., Li Z. (2021). Changes of renal transporters in the kinetic process of VCM-induced nephrotoxicity in mice. Toxicol. Res..

[21] Filippone E.J., Kraft W.K., Farber J.L. (2017). The nephrotoxicity of vancomycin. Clin. Pharmacol. Ther..

[22] Sakamoto Y., Yano T., Hanada Y., Takeshita A., Inagaki F., Masuda S., Matsunaga N., Koyanagi S., Ohdo S. (2017). Vancomycin induces reactive oxygen species-dependent apoptosis via mitochondrial cardiolipin peroxidation in renal tubular epithelial cells. Eur. J. Pharmacol..

[23] Nishino Y., Takemura S., Minamiyama Y., Hirohashi K., Ogino T., Inoue M., Okada S., Kinoshita H. (2003). Targeting superoxide dismutase to renal proximal tubule cells attenuates vancomycin-induced nephrotoxicity in rats. Free Radic. Res..

[24] Fujiwara K., Yoshizaki Y., Shin M., Miyazaki T., Saita T., Nagata S. (2012). Immunocytochemistry for vancomycin using a monoclonal antibody that reveals accumulation of the drug in rat kidney and liver. Antimicrob. Agents Chemother..

[25] Perazella M.A. (2019). Drug-induced acute kidney injury: Diverse mechanisms of tubular injury. Curr. Opin. Crit. Care.

[26] Kwiatkowska E., Domanski L., Dziedziejko V., Kajdy A., Stefanska K., Kwiatkowski S. (2021). The mechanism of drug nephrotoxicity and the methods for preventing kidney damage. Int. J. Mol. Sci..

[27] Bellos I., Pergialiotis V., Perrea D.N. (2021). Kidney biopsy findings in vancomycin-induced acute kidney injury: A pooled analysis. Int. Urol. Nephrol..

[28] Pais G.M., Liu J., Zepcan S., Avedissian S.N., Rhodes N.J., Downes K.J., Moorthy G.S., Scheetz M.H. (2020). Vancomycin-induced kidney injury: Animal models of toxicodynamics, mechanisms of injury, human translation, and potential strategies for prevention. Pharmacotherapy.

[29] Marre R., Schulz E., Anders T., Sack K. (1984). Renal tolerance and pharmacokinetics of vancomycin in rats. J. Antimicrob. Chemother..

[30] Young I.S., Woodside J.V. (2001). Antioxidants in health and disease. J. Clin. Pathol..

[31] Oktem F., Arslan M.K., Ozguner F., Candir O., Yilmaz H.R., Ciris M., Uz E. (2005). In vivo evidences suggesting the role of oxidative stress in pathogenesis of vancomycin-induced nephrotoxicity: Protection by erdosteine. Toxicology.

[32] Lee H.S., Kim S.M., Jang J.H., Park H.D., Lee S.Y. (2021). Serum 5-Hydroxyindoleacetic acid and ratio of 5-hydroxyindoleacetic acid to serotonin as metabolomics indicators for acute oxidative stress and inflammation in vancomycin-associated acute kidney injury. Antioxidants.

[33] Humanes B., Jado J.C., Camano S., Lopez-Parra V., Torres A.M., Alvarez-Sala L.A., Cercenado E., Tejedor A., Lazaro A. (2015). Protective effects of cilastatin against vancomycin-induced nephrotoxicity. Biomed Res. Int..

[34] King D.W., Smith M.A. (2004). Proliferative responses observed following vancomycin treatment in renal proximal tubule epithelial cells. Toxicol. Vitr..

[35] Heller B., Wang Z.Q., Wagner E.F., Radons J., Burkle A., Fehsel K., Burkart V., Kolb H. (1995). Inactivation of the poly(ADP-ribose) polymerase gene affects oxygen radical and nitric oxide toxicity in islet cells. J. Biol. Chem..

[36] Wang J., Li H., Qiu S., Dong Z., Xiang X., Zhang D. (2017). MBD2 upregulates miR-301a-5p to induce kidney cell apoptosis during vancomycin-induced A.K.I. Cell Death Dis..

[37] Xu X., Pan J., Li H., Li X., Fang F., Wu D., Zhou Y., Zheng P., Xiong L., Zhang D. (2019). Atg7 mediates renal tubular cell apoptosis in vancomycin nephrotoxicity through activation of PKC-delta. FASEB J..

[38] Jiang M., Wei Q., Dong G., Komatsu M., Su Y., Dong Z. (2012). Autophagy in proximal tubules protects against acute kidney injury. Kidney Int..

[39] Boya P., Kroemer G. (2008). Lysosomal membrane permeabilization in cell death. Oncogene.

[40] Pannu N., Nadim M.K. (2008). An overview of drug-induced acute kidney injury. Crit. Care Med..

[41] Hosohata K. (2016). Role of oxidative stress in drug-induced kidney injury. Int. J. Mol. Sci..

[42] Gelfand M.S., Cleveland K.O., Mazumder S.A. (2014). Vancomycin-induced interstitial nephritis superimposed on coexisting renal disease: The importance of renal biopsy. Am. J. Med. Sci..

[43] Azar R., Bakhache E., Boldron A. (1996). Acute interstitial nephropathy induced by vancomycin. Nephrologie.

[44] Perazella M.A., Markowitz G.S. (2010). Drug-induced acute interstitial nephritis. Nat. Rev. Nephrol..

[45] Htike N.L., Santoro J., Gilbert B., Elfenbein I.B., Teehan G. (2012). Biopsy-proven vancomycin-associated interstitial nephritis and acute tubular necrosis. Clin. Exp. Nephrol..

[46] Dieterich C., Puey A., Lin S., Swezey R., Furimsky A., Fairchild D., Mirsalis J.C., Ng H.H. (2009). Gene expression analysis reveals new possible mechanisms of vancomycin-induced nephrotoxicity and identifies gene markers candidates. Toxicol. Sci..

[47] Davies S.W., Efird J.T., Guidry C.A., Dietch Z.C., Willis R.N., Shah P.M., Sawyer R.G. (2016). Top guns: The “Maverick” and “Goose” of empiric therapy. Surg. Infect..

[48] Schreier D.J., Kashani K.B., Sakhuja A., Mara K.C., Tootooni M.S., Personett H.A., Nelson S., Rule A.D., Steckelberg J.M., Tande A.J. (2019). Incidence of acute kidney injury among critically ill patients with brief empiric use of antipseudomonal beta-lactams with vancomycin. Clin. Infect. Dis..

[49] Yi Y.H., Wang J.L., Yin W.J., Xu W.H. (2021). Vancomycin or daptomycin plus a beta-lactam versus vancomycin or daptomycin alone for methicillin-resistant staphylococcus aureus bloodstream infections: A systematic review and meta-analysis. Microb. Drug. Resist..

[50] Luther M.K., Timbrook T.T., Caffrey A.R., Dosa D., Lodise T.P., LaPlante K.L. (2018). Vancomycin plus piperacillin-tazobactam and acute kidney injury in adults: A systematic review and meta-analysis. Crit. Care Med..

[51] Bellos I., Karageorgiou V., Pergialiotis V., Perrea D.N. (2020). Acute kidney injury following the concurrent administration of antipseudomonal β-lactams and vancomycin: A network meta-analysis. Clin. Microbiol. Infect. Off. Publ. Eur. Soc. Clin. Microbiol. Infect. Dis..

[52] Kim T., Kandiah S., Patel M., Rab S., Wong J., Xue W., Easley K., Anderson A.M. (2015). Risk factors for kidney injury during vancomycin and piperacillin/tazobactam administration, including increased odds of injury with combination therapy. BMC Res. Notes.

[53] Jensen J.U., Hein L., Lundgren B., Bestle M.H., Mohr T., Andersen M.H., Thornberg K.J., Loken J., Steensen M., Fox Z. (2012). Kidney failure related to broad-spectrum antibiotics in critically ill patients: Secondary end point results from a 1200 patient randomised trial. BMJ Open.

[54] Elyasi S., Khalili H., Dashti-Khavidaki S., Mohammadpour A. (2012). Vancomycin-induced nephrotoxicity: Mechanism, incidence, risk factors and special populations. A literature review. Eur. J. Clin. Pharmacol..

[55] Burgess L.D., Drew R.H. (2014). Comparison of the incidence of vancomycin-induced nephrotoxicity in hospitalized patients with and without concomitant piperacillin-tazobactam. Pharmacotherapy.

[56] Avedissian S.N., Pais G.M., Liu J., Rhodes N.J., Scheetz M.H. (2020). Piperacillin-tazobactam added to vancomycin increases risk for acute kidney injury: Fact or fiction?. Clin. Infect. Dis. Off. Publ. Infect. Dis. Soc. Am..

[57] Vaidya V.S., Ozer J.S., Dieterle F., Collings F.B., Ramirez V., Troth S., Muniappa N., Thudium D., Gerhold D., Holder D.J. (2010). Kidney injury molecule-1 outperforms traditional biomarkers of kidney injury in preclinical biomarker qualification studies. Nat. Biotechnol..

[58] Aljefri D.M., Avedissian S.N., Rhodes N.J., Postelnick M.J., Nguyen K., Scheetz M.H. (2019). Vancomycin area under the curve and acute kidney injury: A meta-analysis. Clin. Infect. Dis..

[59] Flannery A.H., Bissell B.D., Bastin M.T., Morris P.E., Neyra J.A. (2020). Continuous versus intermittent infusion of vancomycin and the risk of acute kidney injury in critically ill adults: A systematic review and meta-analysis. Crit. Care Med..

[60] O’Donnell J.N., Rhodes N.J., Lodise T.P., Prozialeck W.C., Miglis C.M., Joshi M.D., Venkatesan N., Pais G., Cluff C., Lamar P.C. (2017). 24-hour pharmacokinetic relationships for vancomycin and novel urinary biomarkers of acute kidney injury. Antimicrob. Agents Chemother..

[61] Drouet M., Chai F., Barthelemy C., Lebuffe G., Debaene B., Decaudin B., Odou P. (2015). Influence of vancomycin infusion methods on endothelial cell toxicity. Antimicrob. Agents Chemother..

[62] Pais G.M., Avedissian S.N., O’Donnell J.N., Rhodes N.J., Lodise T.P., Prozialeck W.C., Lamar P.C., Cluff C., Gulati A., Fitzgerald J.C. (2019). Comparative performance of urinary biomarkers for vancomycin-induced kidney injury according to timeline of injury. Antimicrob. Agents Chemother..

[63] Pang H.M., Qin X.L., Liu T.T., Wei W.X., Cheng D.H., Lu H., Guo Q., Jing L. (2017). Urinary kidney injury molecule-1 and neutrophil gelatinase-associated lipocalin as early biomarkers for predicting vancomycin-associated acute kidney injury: A prospective study. Eur. Rev. Med. Pharmacol. Sci..

[64] Sampaio de Souza Garms D., Cardoso Eid K.Z., Burdmann E.A., Marcal L.J., Antonangelo L., Dos Santos A., Ponce D. (2021). The role of urinary biomarkers as diagnostic and prognostic predictors of acute kidney injury associated with vancomycin. Front. Pharmacol..

[65] Kane-Gill S.L., Ostermann M., Shi J., Joyce E.L., Kellum J.A. (2019). Evaluating renal stress using pharmacokinetic urinary biomarker data in critically ill patients receiving vancomycin and/or piperacillin-tazobactam: A secondary analysis of the multicenter sapphire study. Drug Saf..

[66] Kim S.M., Lee H.S., Kim M.J., Park H.D., Lee S.Y. (2021). Diagnostic value of multiple serum biomarkers for vancomycin-induced kidney injury. J. Clin. Med..

[67] Yang X., Zhong H., Xu C., Xu G. (2019). Spotlights on antibiotic-induced acute kidney injury: The evidence to date. Iran. J. Kidney Dis..

[68] Zonozi R., Wu A., Shin J.I., Secora A., Coresh J., Inker L.A., Chang A.R., Grams M.E. (2019). Elevated vancomycin trough levels in a tertiary health system: Frequency, risk factors, and prognosis. Mayo Clin. Proc..

[69] Watkins R.R., Deresinski S. (2017). Increasing evidence of the nephrotoxicity of piperacillin/tazobactam and vancomycin combination therapy-what is the clinician to do?. Clin. Infect. Dis..

[70] Rybak M.J., Le J., Lodise T.P., Levine D.P., Bradley J.S., Liu C., Mueller B.A., Pai M.P., Wong-Beringer A., Rotschafer J.C. (2020). Therapeutic monitoring of vancomycin for serious methicillin-resistant Staphylococcus aureus infections: A revised consensus guideline and review by the American Society of Health-System Pharmacists, the Infectious Diseases Society of America, the Pediatric Infectious Diseases Society, and the Society of Infectious Diseases Pharmacists. Am. J. Health Syst. Pharm..

[71] Rossert J. (2001). Drug-induced acute interstitial nephritis. Kidney Int..

[72] Wicklow B.A., Ogborn M.R., Gibson I.W., Blydt-Hansen T.D. (2006). Biopsy-proven acute tubular necrosis in a child attributed to vancomycin intoxication. Pediatr. Nephrol..

[73] DeSoi C.A., Sahm D.F., Umans J.G. (1992). Vancomycin elimination during high-flux hemodialysis: Kinetic model and comparison of four membranes. Am. J. Kidney Dis..

[74] Bamgbola O. (2016). Review of vancomycin-induced renal toxicity: An update. Ther. Adv. Endocrinol. Metab..

